# A clinical study of the LiVac laparoscopic liver retractor system

**DOI:** 10.1007/s00464-015-4272-0

**Published:** 2015-06-20

**Authors:** Philip Gan, Judy Bingham

**Affiliations:** St John of God Hospital, Suite 7, 136 Botanic Road, Warrnambool, VIC 3280 Australia; Easington Pty Ltd, P.O. Box 1201, Camberwell, VIC 3124 Australia

**Keywords:** Laparoscopic, Liver retraction, Vacuum, Suction

## Abstract

**Background:**

All retractors for laparoscopic operations on the gallbladder or stomach apply an upward force to the under-surface of the liver or gallbladder, most requiring an additional skin incision. The LiVac laparoscopic liver retractor system (LiVac retractor) comprises a soft silicone ring attached to suction tubing and connected to a regulated source of suction. The suction tubing extends alongside existing ports. When placed between the liver and diaphragm, and suction applied, a vacuum is created within the ring, keeping these in apposition. Following successful proof-of-concept animal testing, a clinical study was conducted to evaluate the performance and safety of the retractor in patients.

**Methods:**

The study was a dual-centre, single-surgeon, open-label study and recruited ten patients scheduled to undergo routine upper abdominal laparoscopic surgery including cholecystectomy, primary gastric banding surgery or fundoplication. The study was conducted at two sites and was approved by the institutions’ ethics committees. The primary objective of the study was to evaluate the performance of the LiVac retractor in patients undergoing upper abdominal single- or multi-port laparoscopic surgery. Performance was measured by the attainment of milestones for the retractor and accessory bevel, where used, and safety outcomes through the recording of adverse events, physical parameters, pain scales, blood tests and a post-operative liver ultrasound.

**Results:**

The LiVac retractor achieved both primary and secondary performance and safety objectives in all patients. No serious adverse events and no device-related adverse events or device deficiencies were reported.

**Conclusion:**

The LiVac retractor achieved effective liver retraction without clinically significant trauma and has potential application in multi- or single-port laparoscopic upper abdominal surgery. As a separate incision is not required, the use of the LiVac retractor in multi-port surgery therefore reduces the number of incisions.

Upward retraction of either lobe of liver is required for surgical access to the gallbladder or stomach in laparoscopic surgery, most commonly through the insertion of a laparoscopic grasper or retractor through a laparoscopic port (fan retractor, snake retractor) or incision (Nathanson liver retractor, Iron Intern), which are all forms of external retractors.

New techniques in laparoscopic surgery have been developed over time to reduce the number of incisions required, leading to reduced-port, single-port laparoscopic surgery and Natural Orifice Translumenal Endoscopic Surgery (NOTES) [1]. Internal retractors have been developed which typically involve a band or tape running between two anchoring mechanisms that are internally attached (Aesculap Cinch, VersaLifter/Band, EndoGrab™/EndoLift™), and which either push up against the liver or grasp the gallbladder. Retraction techniques may also involve the passage of sutures through the full thickness of liver and abdominal wall or suturing the gallbladder [1–3]. Other retraction devices also involve suturing through the abdominal wall (A.M.I. EndoSail, Disc suspension [4]). All these devices or techniques apply a force to the liver from below. Cyanoacrylate glue has been reported to adhere the liver to the diaphragm, [5] but this technique would arguably be unlikely to gain traction given the permanent nature of the adhesion and the implications should the patients ever require liver surgery in future.

The LiVac laparoscopic liver retractor system (LiVac retractor) is a novel laparoscopic liver retractor comprising a disposable, soft, collapsible silicone ring-shaped device connected to suction tubing. The LiVac retractor is placed between the liver and diaphragm, and suction is then applied to the tubing, which then apposes the liver and diaphragm with vacuum forces [6]. The name LiVac is derived from liver vacuum.

A first-in-human study was conducted to evaluate the performance and safety of this retractor in a range of single- and reduced-port laparoscopic cholecystectomy and gastric operations.

## Materials and methods

**Fig. 1:**
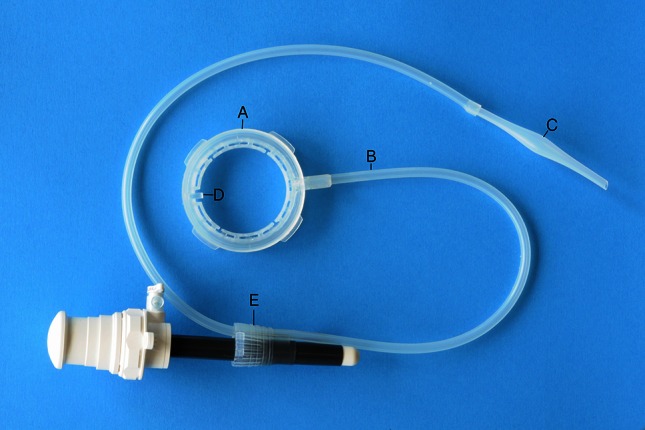
LiVac laparoscopic liver retractor system

The LiVac retractor, shown in Fig. 1, is a soft silicone ring (A) connected to suction tubing (B). The suction tubing connects to a large calibre external (sterile) suction hose via a connector (C). The suction hose in turn is connected to a suction canister, high-pressure regulator and the operating theatre wall suction. A high-pressure regulator is mandatory to control the vacuum forces.

The LiVac retractor is grasped at slot (D), lubricated and inserted into the abdominal cavity using an O’Brien inserter or laparoscopic grasper.

The LiVac retractor can be used as a stand-alone device, or in conjunction with the accessory LiVac bevel (E), which replaces the bevel (cone) in a Hasson-type port and allows the tubing to exit the abdomen without carbon dioxide leak.

Two sizes of LiVac retractor were used: small (56 mm diameter) and large (80 mm diameter). The small retractors were used for all left lobe retractions, and a mixture of small and large retractors were used for the right lobe retractions (cholecystectomies).

During surgery, LiVac retractor was inserted directly through the surgical incision for the single-port and reduced-port cholecystectomies and through the lumen of the 15-mm optical ports, which were used for the gastric banding and fundoplication operations. The accessory LiVac bevel was only used for reduced-port cholecystectomies.

### Study approval

The study was approved by the institutional Human Research Ethics Committees, which are constituted under the Australian National Health and Medical Research Council (NHMRC) Guidelines [7]. The study was conducted in accordance with the Australian Clinical Trials Notification Scheme [8] and ISO 14155: 2011 [9]. Prior to enrolment in the study, all patients received information about the study and signed written informed consent.

The study was registered on the Australian and New Zealand Clinical Trials Registry (ANZCTR) [10].

### Patient selection/exclusion criteria

The study was a multi (two)-centre open-label non-randomised first-in-human study to evaluate the LiVac retractor in patients scheduled to undergo routine elective upper abdominal laparoscopic surgery including cholecystectomy, primary gastric banding surgery or fundoplication. Inclusion criteria included ages 18–65, ASA I or II, [11] and competence to consent. Major exclusion criteria included patients undergoing emergency procedures, patients with chronic liver disease and significant co-morbidities.

### Objectives

The primary objective was to evaluate the performance of the LiVac retractor in attaining retraction of the liver such that the intended surgery could be completed. Secondary objectives included evaluation of the safety and tolerability of the retractor and the performance of the accessory LiVac bevel. Exploratory objectives included evaluation of the number of times the retractor was re-positioned and the vacuum pressure settings and changes required. The lobe of liver retracted and size of port used were also recorded.

### Performance measures

Performance measures (milestones) were used to assess the functioning of the retractor and bevel as shown in Table 1.

**Table 1:** Performance milestones

Milestone	LiVac retractor	LiVac bevel (Hasson port procedures only)
1	Device inserted correctly into the peritoneum	Device inserted correctly into the peritoneum
2	Adequate seal obtained	Adequate stay sutures and LiVac bevel seal obtained
3	Retraction of liver (record suction pressure)	Connection to external suction tubing and suction retraction initiated
4	Adequate vision of underlying organs, particularly stomach and gallbladder	Not applicable
5	Sustained retraction of liver: planned surgery able to proceed.	Suction retraction sustained
6	Sustained retraction of liver: surgery complete	Not applicable
7	Successful conclusion of retraction in vivo (device turned off)	Suction retraction ceased and retractor disengaged without significant trauma
8	Successful withdrawal of LiVac retractor through incision	Successful withdrawal of LiVac bevel

### Safety assessments

Safety outcome measures included the recording of adverse events, safety laboratory assessments [electrolyte and liver function biochemistry, haematology, coagulation], blood loss during procedure, physical examinations, vital signs, post-operative pain score and post-operative liver ultrasound.

### Data collection

All data in the Case Report Forms (CRFs) were entered independently by Clinical Trials Coordinators, who attended each surgical procedure. All surgical procedures were video-recorded, and each patient had a liver ultrasound on day 1 after surgery. The study was independently monitored, and the data were independently analysed and reported. An independent surgeon was available to review any serious adverse events, related adverse events or device deficiencies.

## Results

Ten patients were enrolled in the study, and all patients completed all six study visits. All patients were considered assessable. Patient ages ranged from 35 to 65 and included nine females and one male. Average BMI was 32.3 (24–41) Table 2.

**Table 2:** Subject demographics and surgical procedure

Study subject ID	Age	Gender	BMI	Type of surgery
1	44	F	31	Three-port hiatus hernia repair and fundoplication
2	44	F	29	SILS™ cholecystectomy
3	43	F	29	SILS™ cholecystectomy
4	54	F	32	Three-port cholecystectomy
5	52	F	29	Three-port cholecystectomy
6	35	F	29	SILS™ cholecystectomy
7	41	F	41	Three-port laparoscopic gastric banding
8	60	M	40	Three-port laparoscopic gastric banding
9	65	F	24	Three-port cholecystectomy
10	38	F	39	Three-port laparoscopic gastric banding

**Fig. 2:**
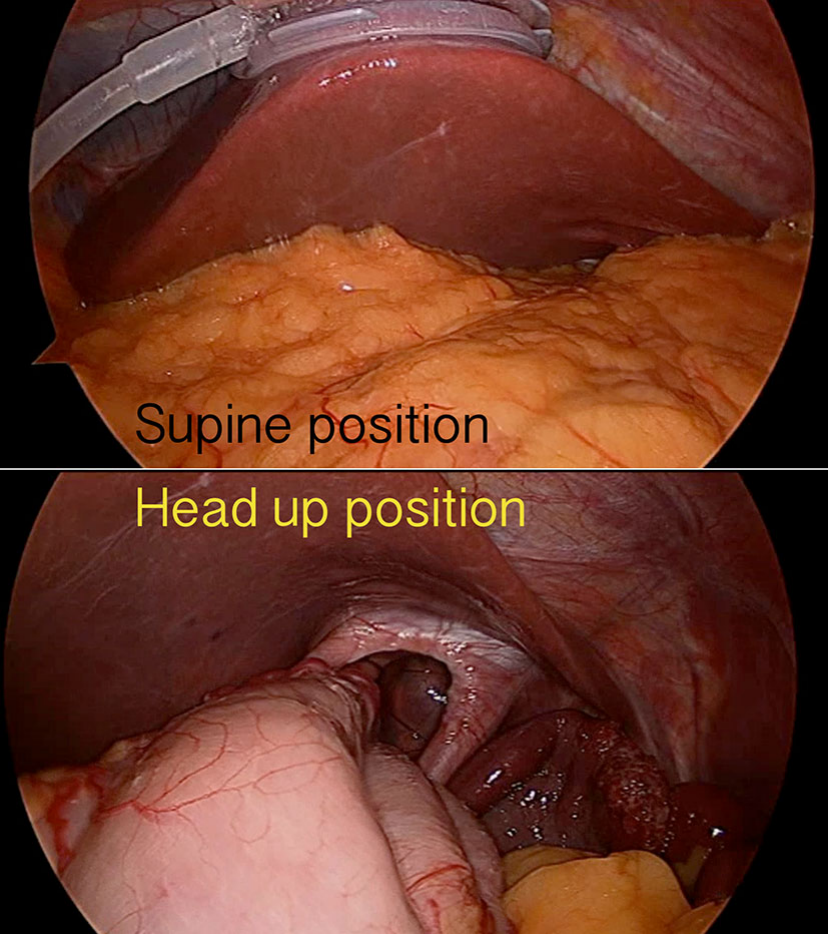
LiVac retraction for fundoplication

**Fig. 3:**
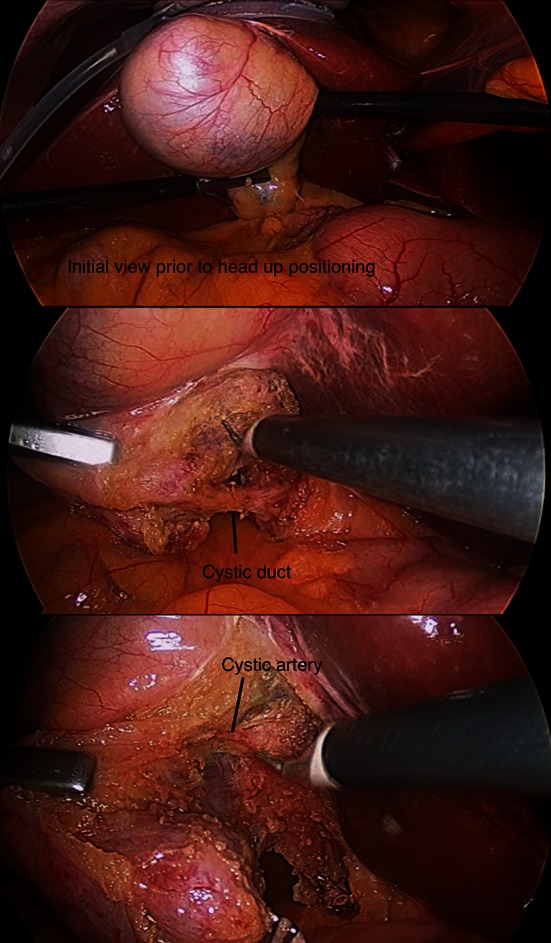
LiVac retraction for three-port cholecystectomy

Surgical procedures included one reduced (three)-port laparoscopic repair of intra-thoracic hiatus hernia with anterior fundoplication (Fig. 2), three reduced (three)-port cholecystectomies (Fig. 3), three SILS™ cholecystectomies and three reduced (three)-port laparoscopic gastric bands. All cholecystectomy patients had an intra-operative cholangiogram. The LiVac bevel (Hasson) was used in the reduced (three)-port cholecystectomies, the Covidien SILS™ port for the SILS™ cholecystectomies, and the Applied Medical 15-mm Kii Optical Access System port was used for the laparoscopic adjustable gastric banding (MIDBAND™) operations.

As shown in Table 3, there were no observed device deficiencies and no functional failures of the LiVac retractor or LiVac bevel. There were no serious adverse events reported and no adverse events relating to the use of the device. All milestones were achieved.

**Table 3:** Retractor and bevel performance

Subject ID	Pressure change	Reason for change	Final pressure -mmHg	Type of surgery^a^	Size of retractor	Re-position	Reason for change	Bevel used
1	Yes	LiVac positioned over the edge of a small left liver lobe	300	3	S	Yes	To centre the LiVac over the left lobe	No
2	Yes	Leak in external suction canister due to loose connection	300	4	S	Yes	Unintentional	No
3	Yes	Not sealing securely at lower pressure setting	400	4	L	Yes	Unintentional	No
4	No		400	1	L	No		Yes
5	No		450	1	L	No		Yes
6	Yes	External suction hose not tightened sufficiently. Identified early	400	4	L	No		No
7	No		320	2	S	No		No
8	No		470	2	S	No		No
9	No		330	1	L	No		Yes
10	No		280	2	S	No		No

^a^1 = reduced-port cholecystectomy, 2 = gastric banding, 3 = fundoplication, 4 = SILS™ cholecystectomy

One patient (No. 1) had a very small left lobe of liver. At the first positioning attempt, the device was overlapping the edge of the liver lobe. An adequate seal was obtained following repositioning of the LiVac retractor.

In three patients (Nos. 2, 3, and 6), retraction failed initially due to a lower suction pressure setting, leak in an external suction canister and the connector not being pushed firmly into the suction hose, respectively. These events were unrelated to the LiVac device itself. For all the subsequent operations, the suction canisters were checked for loose connections, and there were no further problems.

The overall duration of surgery as shown in Table 4 ranged from 34 to 134 min, whilst the duration of use of the LiVac retractor ranged from 11 to 100 min, with an average duration of 35 min. The longest procedure was a fundoplication in which the LiVac retractor was used for 100 min. The duration of retraction averaged 22 min for reduced-port cholecystectomies, 32 min for SILS™ cholecystectomies and 28 min for gastric banding procedures.

**Table 4:** Duration of surgery and use of LiVac retractor

Procedure	Number of procedures	Duration of surgery (minutes)		Duration of use of LiVac retractor (minutes)	
		Range	Average	Range	Average
Three-port cholecystectomy	3	48–64	57	19–25	22
SILS™ cholecystectomy	3	62–79	72	31–34	32
Gastric banding	3	34–77	52	11–49	28
Fundoplication	1	134	134	100	100
Total	10	34–134	68	11–100	35

**Fig. 4:**
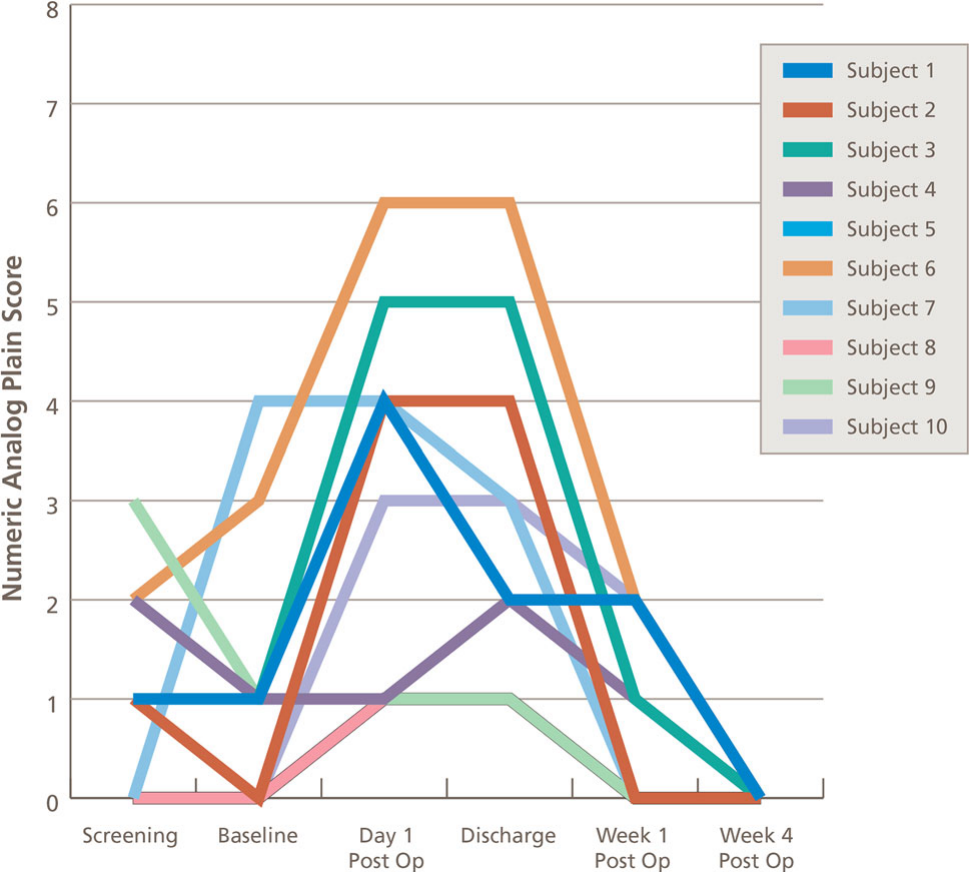
Pain scores

Pain was assessed in all patients at all study visits, using the numeric rating scale, where 0 = no pain and 10 = worst pain imaginable, which are recorded in Fig. 4. There was no comparator for pain scores in this small study. Nine patients went home on day 1 after surgery, and the patient with intra-thoracic hiatus hernia went home on day 3.

**Fig. 5:**
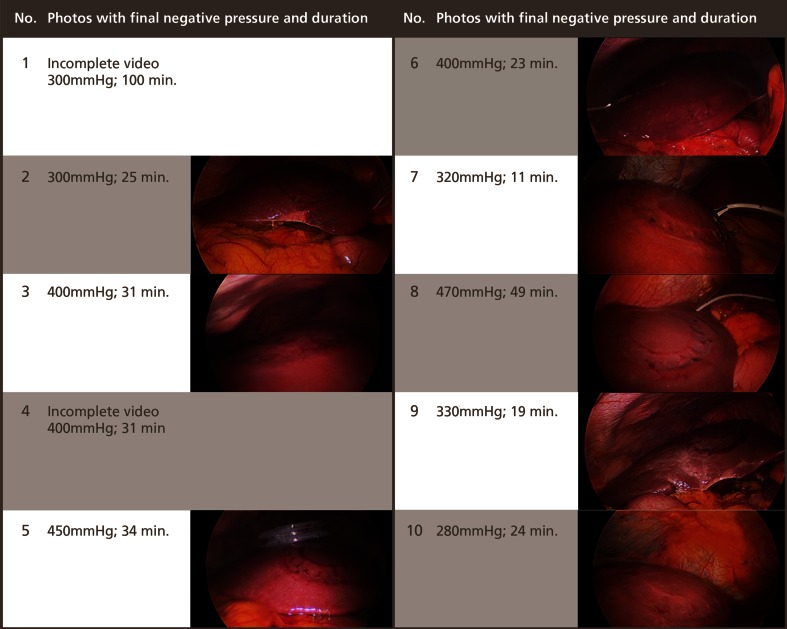
Liver images following LiVac Retraction

The appearance of the liver post-retraction was recorded by the attending Clinical Trials Coordinator. No bleeding, serosal tears or lacerations were observed. Consistent with observations during the pre-clinical animal trials, initial embossing of the liver occurred, which flattened, leaving only bruising. Due to a 4-GB recording limit setting, the operation recordings were incomplete for the patients Nos 1 and 4. Following technical advice, resetting the limit to 20 GB, recordings for all subsequent patients were complete. The post-retraction images are demonstrated in Fig. 5.

At screening, all blood test results were within normal range, with the exception of two patients. Patient No. 8 had mildly elevated liver function tests, consistent with his known fatty liver, BMI 40 and waist/hip ratio of 1.1. Patient No. 10 had mildly elevated ALP at screening. AST measurements are shown in Table 5 (bold values outside of normal reference range).

**Table 5:** AST measurements

Subject	Operation	Pre-operative	Day 1 postoperative	Week 1 postoperative	Follow-up
1	Fundoplication	26	27	**42**	Normal at week 4
2	Cholecystectomy	18	**54**	34	
3	Cholecystectomy	18	19	30	
4	Cholecystectomy	24	25	32	
5	Cholecystectomy	19	19	27	
6	Cholecystectomy	13	18	16	
7	Gastric band	ND*	22	**49**	Normal at week 3
8	Gastric band	**42**	42	**50**	
9	Cholecystectomy	31	40	**54**	
10	Gastric band	16	Not available	40	

Three of the four patients with elevated AST at week 1 were on the Optifast^®^ liquid meal replacement programme as was the surgeon’s routine following fundoplication and gastric banding operations. Transient elevation of hepatic enzymes is reported to occur during treatment with Optifast [12]. These patients had normal AST measurements (<41 U/L) on day 1 post-operatively.

## Discussion

The LiVac retractor is simple in concept and in use, with a minimal learning curve as exemplified by the successful application in each of these first ten patients. The use of this retractor does not fundamentally change the techniques required for the operations in which it is used, but requires fewer incisions (reduced port). In single-port cholecystectomy, liver retraction was attained without suturing the gallbladder, placing intercostal sutures or using a hand-held grasper, which increases hand clashing. As the liver itself is retracted when using the LiVac retractor in both reduced-port and single-port cholecystectomy, the plane between the liver and gallbladder opens up during dissection. The surgical assistant no longer holds a retractor and can focus on directing the laparoscope, with the other hand free to readily change the orientation of the angled scope, potentially improving exposure.

All currently available liver retractor devices or methods of retraction carry some risk of liver injury. The Nathanson retractor has been in common use for decades and is recognised to cause congestion of the liver through compression of the liver parenchyma and associated vasculature. Liver haematoma, hepatic necrosis and atrophy have been reported with the Nathanson retractor [13–16]. It is, however, a strong retractor, being made of steel and fixed to an external frame. As the LiVac retractor is the only liver retractor that attaches to the superior surface and does not push up against the liver parenchyma, there is no compression of the tissue or vessels. Hand-held retractors carry the advantage of manoeuvrability, but are dependent upon an appropriately skilled assistant and carry a risk of fatigue. The assistant, not the surgeon, also determines the amount of force applied to the liver by hand-held retractors. Completely internal forms of retraction require fixation between two sites using clamps or hooks, with the band, suture or rod between these two points of fixation pushing up against the liver in a linear manner. The liver nevertheless tends to drape on either side of the retractor.

The diaphragm and liver conform to the internal contours of the LiVac retractor as they are drawn into the ring under vacuum. The surface embossing seen as the LiVac retractor was released completely flattened out within minutes, and there was no bleeding or serosal laceration. Furthermore, all patients had a normal liver ultrasound on post-operative day 1 and AST changes were insignificant. Only a limited depth of tissue is drawn into the device, and this appears to therefore limit the depth of associated haemorrhagic changes. These results were consistent with earlier testing in animals, in which liver histology had been obtained. In a study on two sheep, which were recovered and then killed on post-operative day 5, histological evidence of trauma was limited to the serosa only, with normal underlying liver parenchyma [6]. Similarly, in porcine testing where the liver was retracted in the same location for a total of 97 min at −400 mmHg (unpublished data) and immediately resected, the pathologist reported that histological evidence of trauma in the liver resected immediately post-procedure “*did not extend beyond 1*–*2* *mm beyond the capsular surface*”. Safety of the LiVac retractor has therefore been demonstrated to be equal if not superior to existing methods.

## Conclusion

The LiVac retractor achieved effective liver retraction without clinically significant trauma and has potential application in a wide range of multi- or single-port laparoscopic upper abdominal surgery. As a separate incision is not required, the use of the LiVac retractor in multi-port surgery therefore reduces the number of incisions.
